# Supplementary material for: Are carbon emissions trading and green financial instruments synergistic? -Comprehensive quantitative research based on content analysis
Source: PLoS One. 2024 Mar 7;19(3):e0298601. doi: 10.1371/journal.pone.0298601 (PMC10919601; doi:10.1371/journal.pone.0298601)
Supplement: S1 Data — (ZIP) [file pone.0298601.s001.zip › Data/地级市Z_F工作报告.zip!/║╙▒▒/╠╞╔╜╩╨/└┤╫╘╣1⁄2╓┌║┼í╛┬φ┐╦╩2╛▌═°í┐_2006.docx]

一、“十五”时期全市经济社会发展的回顾

过来五年，是我市在全面建设小康社会道路上阔步前进、跨越发展的五年。在市委的正确领导下，全市人民坚持以邓小平理论和“三个代表”重要思想为指导，树立和落实科学发展观，全面贯彻党的十六大精神和中央、省一系列决策部署，沉着应对前进道路上的各种严峻考验，抢抓机遇，埋头苦干，较好地完成了“十五”计划目标，经济社会发展取得了重大成就。

五年来，我们坚持狠抓发展第一要务，加快经济结构战略性调整，国民经济实力跨上一个大台阶。2005年预计全市生产总值突破2000亿元大关，“十五”年均增长12.7%，比预期目标高2.7个百分点;其中，一、二、三产年均分别增长4.9%、15.1%和12.5%。人均生产总值达到2.8万元。全部财政收入226亿元，年均增长36.3%。农业和农村经济全面发展。全面落实各项惠农政策，粮食生产稳定增长。乳业、瘦肉型猪、果菜、板栗、花生、水产品六大龙型经济形成规模，农业产业化经营率达到55%。畜牧水产业占农业产值的比重上升到51%，成为农业第一大主导产业。转移农村富余劳动力71.2万人次。农村税费改革不断深化，全市农民人均负担比“九五”末下降85%。工业结构调整取得明显成效。2005年全市规模以上工业预计完成增加值800亿元、利税265亿元，分别比“九五”末增长1.5倍和2.8倍。钢铁、建材、能源、装备制造和化工五大优势产业竞争实力显著增强，增加值、利税分别占全市规模以上工业的85%以上。积极有效地贯彻落实国家宏观调控政策，钢铁产业整合调整取得重要进展。全市钢铁冶炼企业由“九五”末的195家减少到30家，板带比达到57%。陶瓷、食品、医药、纺织服装等传统产业发展水平不断提升。高新技术产业群体初步形成。服务业稳步发展。2005年全社会消费品零售总额预计达到410亿元，“十五”年均增长12.1%。商贸流通、邮电通信、餐饮服务等传统产业水平和档次明显提高，交通运输形成海陆一体化格局,房地产、旅游、信息等现代服务业发展步伐加快。项目建设和县域经济支撑作用显著增强。五年间，全社会固定资产投资累计完成1858亿元，是“九五”的1.9倍。其中，重点项目累计完成投资493亿元，以“四大兴市工程”为代表的一大批重点项目陆续建成，形成了新的经济增长群。县域经济实力不断壮大。2005年全市县域生产总值、财政收入分别占到全市的70%和52%。迁安、遵化跨入全国百强行列。

五年来，我们坚持实施以港兴市战略，生产力布局向沿海推进取得重大突破，为未来跨越式发展提供了战略支撑。唐山港京唐港区功能进一步完善，年货物吞吐量突破3300万吨;曹妃甸港区纳入国家渤海湾地区港口建设规划，建成2座25万吨级矿石码头并实现国际通航。曹妃甸工业区大规模开发建设全面展开。通路、通信、供电、供水和围海造地等基础设施建设配套推进。首钢京唐钢铁联合有限公司正式组建。迁曹铁路开工建设。曹妃甸工业区总体规划和专项规划取得阶段性成果。曹妃甸循环经济示范区被列入国家循环经济试点园区。海港开发区和南堡开发区相继有一大批重点项目投入建设，经济社会发展加速。沿海经济隆起带已具雏形。

五年来，我们坚持推进以国有企业为重点的各项改革，大力发展民营经济，扩大双向开放，经济发展活力显著增强。以国有企业为重点的各项改革取得明显成效。市、县(市)区属1154家国有企业全部完成改制，累计退出国有资本211.5亿元。135家国有及国有控股企业建立起法人治理结构基本框架。上市公司发展到7家。省市属企业及石油、铁路系统分离企业办社会职能顺利完成。下岗职工基本生活保障与失业保险顺利并轨。国有资产监管体系进一步健全。行政管理体制改革取得明显成效。事业单位改革稳步推进。要素市场健康发展。流通、财税金融、投融资等各项改革不断深化。民营经济迅猛发展。增加值占全市生产总值的比重达到57%，民营经济从业人员由“九五”末的94.4万人增加到133.8万人。对外开放规模和水平进一步提高。“十五”期间实际利用外资13.7亿美元，是我市对外开放以来利用外资最多的时期。一批外资工业园初具规模。对外贸易迅速增长。2005年全市进出口总额达到26亿美元，其中出口11.5亿美元，分别比“九五”末增长4.3倍和2.3倍。外经工作领域和规模进一步扩大。区域经济合作特别是与北京市的合作取得重大成果。五年累计引进市外资金305亿元。成功举办第四至第八届唐山中国陶瓷博览会，活跃了开放工作全局。

五年来，我们坚持贯彻科学发展观，统筹推进城乡、经济社会和人与自然协调发展，促进了社会全面进步。城市化进程明显加快。全市城市化水平达到45%，比“九五”末提高9.7个百分点。新一轮城市总体规划纲要修编通过国家建设部评审。丰南撤市建区，丰润、新区合并建区;芦台、汉沽分别改建开发区和管理区;曹妃甸组建工业区;市区南部采沉区生态建设和机场新区建设全面启动，形成了新的城市发展格局。“十五”期间，累计投资近500亿元。相继建成了污水处理、城市供水、集中供热、煤气输配等一批重点市政工程;新建住宅860万平方米;新建改造城市道路58条;新建、改建国省干线公路293公里;城市建成区绿化覆盖率由“九五”末的38.4%提高到43.9%。县城和小城镇建设成为我市经济社会发展中的一大亮点，文明生态村建设深入推进，城乡面貌显著改观。各项社会事业长足发展。市县财政投入社会事业发展的资金达137.2亿元，年均递增24.1%。“科教兴市”战略深入实施。科技创新能力不断增强，科技进步对经济增长的贡献率达到56%。义务教育水平得到巩固提高，素质教育扎实推进;中小学布局进一步优化，农村中小学危房改造全部完成，高中阶段毛入学率由“九五”末的59%提高到78.5%;高等教育形成了“四本多专”的新格局;人才培养、引进取得新成效，人才队伍不断壮大。文化事业繁荣发展。群众文化活动蓬勃开展，一批文艺作品获奖，成功举办了第二至第四届中国评剧艺术节和2005中国国际皮影艺术展演，冀东优秀文化进一步发扬光大。城乡医疗卫生水平明显提高。抗击“非典”斗争取得重大胜利;建立健全了疾病预防控制、医疗救治、卫生执法监督和疫情监测体系;加强农村卫生基础设施建设，对135所乡镇卫生院全部进行了改造扩建;迁安、丰南新型农村合作医疗试点工作取得明显成效。全民健身活动广泛开展，竞技体育水平不断提高。广播电视、民政、民族宗教、外事侨务、爱国卫生、防震减灾、气象、档案、人防、民兵预备役和国防动员等各项工作也都取得了新的成绩。人口、资源、环境工作得到加强。全市计划生育率保持在98%以上，人口自然增长率3.37‰。水、土地、矿产、森林等自然资源保护、节约和合理开发利用取得实效。城乡环境污染得到控制，环境质量进一步改善。2005年城市空气质量二级及优于二级的天数达到290天，为近年来最好水平。

五年来，我们坚持以人为本，下大力解决好就业、社会保障等事关群众利益的热点难点问题，城乡居民生活水平显著提高。预计城镇居民人均可支配收入达到10370元，农民人均纯收入4560元，分别比“九五”末增长52.5%和33.7%。城乡居民消费结构升级，宽带、汽车等现代消费开始进入普通家庭。五年共为群众办实事110件，解决了一大批群众关心的热点难点问题。就业再就业工作收到实效。城镇累计新增就业15万多人，安置下岗职工20.7万人;城镇登记失业率控制在4%以内。独立的社会保障体系基本建立。离退休人员养老金和失业人员失业保险金按时足额发放。城乡最低生活保障制度逐步完善，保障水平不断提高。社会救助体系进一步健全。

五年来，我们坚持物质文明、政治文明、精神文明“三个文明”全面发展的方针，社会主义精神文明取得丰硕成果，民主法制建设不断加强。坚持用邓小平理论和“三个代表”重要思想武装广大干部群众，深入开展保持共产党员先进性教育活动，激发了全市人民加快唐山新世纪发展的热情和干劲。以“创五城”为载体，广泛开展公民道德实践和群众性精神文明创建活动，城乡文明水平和公民素质进一步提高。先后被评为全国创建文明城市工作先进城市、全国双拥模范城、国家园林城市和全国社会治安综合治理优秀城市。坚持并完善了向人大报告工作、向政协通报情况制度，自觉接受人民代表大会及其常务委员会的依法监督，自觉接受人民政协的政治协商、民主监督和参政议政。五年来，承办市以上人大代表批评意见、建议和政协委员提案3474件，促进了政府工作水平的提高。健全完善公众参与、专家咨询、政府决策相结合的决策机制和重要决策失误责任追究制，建立社情民意反映制度，重大事项社会公示、听证、质询制度，提高了政府决策科学化、民主化水平。认真贯彻《行政许可法》，坚持依法治市、依法行政，行政执法责任制和“两错”责任追究制得到较好落实。普法教育不断深入。切实加强人民群众来信来访工作，提高市长公开电话和市长电子信箱办理工作水平，努力为人民群众排忧解难。廉政建设不断加强，反腐败斗争取得阶段性成果。深入开展“诚信平安唐山”建设，强化社会治安综合治理，加强隐蔽战线和与“法轮功”邪教组织的斗争，维护了全市政治安定和社会稳定。

各位代表，随着“十五”计划的全面完成，我市在综合经济实力、产业布局、发展体制、管理机制、投资环境等方面都发生了一系列深刻变化，比较优势和后发优势日益显现，跨越式发展态势已经形成。过去的五年，可以说是我市经济社会发展最快最好的时期。回顾五年来的政府工作，面对错综复杂的宏观环境，我们始终坚持树正气、讲团结、求发展，以“努力走在全省最前列”奋斗目标统一思想，凝聚力量，形成了聚精会神搞建设、一心一意谋发展的强大合力;始终坚持把贯彻落实中央和省的工作部署与唐山实际紧密结合，大胆探索，勇于创新，不断开创改革发展的新局面;始终坚持求真务实的作风，坚韧不拔，锲而不舍，以干大事、创大业的气魄和韧劲，狠抓改革开放、生产力布局和经济结构调整等一系列事关全局的重大问题，带动和活跃了工作全局;始终坚持以人为本、执政为民，关注民本民生，努力促进社会和谐，使改革发展的成果惠及全市人民;始终坚持正确处理改革、发展和稳定的关系，物质文明、政治文明和精神文明一起抓，为全市改革发展创造了良好环境。总之，“十五”期间我市经济和社会发展取得的巨大成绩是来之不易的。这是市委正确领导的结果，是全市人民艰苦奋斗、锐意进取、共同努力的结果，是市人大、市政协监督支持和全市各界人士关心帮助的结果。在此，我代表市政府，向全市工人、农民、知识分子、机关干部、公安政法干警、驻唐部队、武警官兵以及社会各界人士，表示崇高的敬意和衷心的感谢!

回顾过来五年的工作，我们更应注意总结政府工作中的教训、问题和不足，看到前进道路上面临的突出矛盾和困难。主要是：经济结构性矛盾依然比较突出，自主创新能力薄弱，传统产业技术装备和管理水平还较低，钢铁行业生产集中度偏低、调整整合的任务艰巨，服务业发展相对不足;改革开放进程仍显滞后，市场经济体制还不完善，开放型经济竞争力偏低;经济社会协调发展机制还不健全，可持续发展能力有待进一步增强，就业压力仍比较大，社会事业发展相对滞后，城乡居民生活水平差别明显，社会保障体系不够完善;安全生产形势严峻，开平刘官屯煤矿“12.7”矿难给人民群众的生命财产造成重大损失，在社会上产生了严重的负面影响，教训极为沉痛;反腐败斗争和廉政建设任务仍比较艰巨，政府机关自身建设需要进一步加强。对这些问题，我们一定引起高度重视，在今后的工作中认真加以解决。

二、“十一五”时期经济社会发展总体思路和奋斗目标

未来五年，是我市全面建设小康社会进程中承前启后的关键时期。市委七届八次全会在全面分析形势的基础上，作出了我市经济社会发展正处于可以大有作为的“黄金发展期”的科学判断。我们要在这一时期，围绕实现全面建设小康社会和走在全省最前列的奋斗目标，抢抓机遇，迎接挑战，奋力加快发展步伐，把我市现代化建设事业推向一个崭新阶段。

根据市委《关于制定国民经济和社会发展第十一个五年规划的建议》，“十一五”时期我市经济社会发展的总体要求是：坚持以邓小平理论、“三个代表”重要思想为指导，全面落实党的十六届五中全会和省委六届八次全会精神，坚持以经济建设为中心，以科学发展观统领经济社会发展全局，充分发挥支柱产业、经济区位、自然资源和现代化港口的优势，深入实施科教兴市、外向带动、城市化和可持续发展四大主体战略，大力优化经济结构，转变增长方式，推进自主创新，完善体制机制，加快提升综合竞争力，促进人的全面发展，提高人民生活水平，推进文明和谐唐山建设，实现更快更好发展，为提前实现全面建设小康社会、建设现代化沿海大城市的宏伟目标奠定坚实基础。“十一五”时期全市经济和社会发展的主要预期目标是：

(1)国民经济快速健康发展。在优化结构、提高效益和降低消耗的基础上，全市生产总值年均增长13%以上，力争实现比2000年翻两番，人均生产总值突破5万元;全部财政收入年均增长13%左右。

(2)产业结构进一步优化。非农产业比重达到90%以上，现代化农业加快发展，优势主导产业支撑作用显著增强，服务业全面发展，国民经济工业化、现代化水平迈上新台阶。

(3)城市化水平明显提高。城市化率年均提高1.5个百分点，城乡差距逐步缩小，现代化沿海大城市建设取得明显进展。

(4)市场经济体制比较完善。行政管理、社会事业、国有企业、财税金融、社会保障等领域的改革和制度建设取得新突破。开放型经济格局基本形成。五年累计实际利用外资25亿美元; 到“十一五”末进出口总额达到80亿美元，其中出口37亿美元。

(5)资源利用效率显著提高。万元生产总值能耗和水耗分别降低20%，循环经济加快推进，转变增长方式取得明显进展。

(6)可持续发展能力增强。人口年均自然增长率控制在4.5‰以内。城市大气环境质量达到国家空气质量二级标准，水、土地、矿产等重要资源的保护节约与开发利用水平明显提高，局部生态环境恶化趋势得到缓解，人与自然和谐发展。

(7)社会事业协调发展。建成较为完善的科技创新、国民教育、卫生医疗、全民健身等公共服务体系，文化大市建设取得显著成效。

(8)文明和谐社会建设取得明显进展。城市居民人均可支配收入和农民人均纯收入年均分别增长8%和6%。社会就业比较充分，城乡社会保障和公共服务体系更加健全。民主法制和精神文明建设进一步加强，人们生活质量和社会文明程度显著提高。

实现更快更好发展和构建和谐唐山，是我们“十一五”期间面临的两大任务。我们提出上述目标，立足于全面贯彻落实以科学发展观统领经济社会发展全局的根本要求，充分考虑了我市“十一五”规划与2015年实现全面建设小康社会宏伟目标的衔接过渡，充分考虑了未来改革发展的有利条件和不利因素，充分考虑了市情市力和现实基础，是科学求是、积极可靠的。实现上述奋斗目标，必须坚持以经济建设为中心，始终不渝地抓好发展这个第一要务;必须坚持走科学发展之路，转变发展观念，创新发展模式，提高发展质量;必须坚持以人为本，把人均指标作为重要标准，把转变增长方式作为突出任务，把城市化摆在活跃全局的战略位置，把推进城乡协调发展作为工作重点，把改革开放作为发展动力，促进经济和社会协调发展。只要我们坚定信心，科学务实，拼搏进取，就一定能够推动我市综合经济实力跃上一个新台阶，社会发展进入一个新阶段，人民生活提高到一个新水平，使全市人民共享发展成果。

三、全力推进以曹妃甸为核心的沿海经济隆起带建设

曹妃甸港区和曹妃甸工业区的开发建设，是唐山未来发展的战略支撑和经济增长极。“十一五”期间，要坚定不移地贯彻“用蓝色思路改写煤都历史”的指导思想，深入实施“依海强市、以港兴市”战略，以曹妃甸为核心，全力打造沿海经济隆起带，力争在实现经济跨越式发展中再造一个新唐山。到2010年，初步确立沿海经济隆起带的基本框架。

加快曹妃甸工业区的全面开发。以“大港口、大钢铁、大化工、大电能”为目标，举全市之力推进“一号工程”建设。围绕建设循环经济示范区、现代化重化工业基地和国际物流中心的要求，充分发挥曹妃甸地理区位、深水大港、资源组合和产业后发优势，大力构筑钢铁、电力、化工、装备制造和现代物流产业集群;以全新的发展理念，全面推进体制创新、机制创新、技术创新和服务创新，努力把曹妃甸建设成为国际先进、国内一流、高度开放的现代工业区和循环经济示范区。

加快沿海经济隆起带建设。加速生产力布局向沿海推进，生产要素向临港聚集、向园区集中，促进以曹妃甸为核心、以沿海三县(乐亭、滦南、唐海)五区(丰南、海港、南堡、汉沽、芦台)为扇面的沿海经济隆起带迅速崛起。借助曹妃甸工业区、海港开发区和南堡开发区的开发建设，科学规划沿海区域功能定位，加大三县五区的开发开放力度，更好利用沿海区域发展空间，合理摆布重大项目，做大做强临港产业。围绕曹妃甸工业区的四大主导产业，科学安排电力、建材、化工等产业链项目，以及金融、保险、旅游、运输等现代服务业项目，形成集聚效应。积极引导和促进其它县(市)区和相关行业，充分利用曹妃甸开发建设的历史机遇，主动出击，积极参与，开辟新的发展空间，实现与沿海经济隆起带共同发展。

大力发展海洋经济。充分发挥海洋资源优势和潜力，搞好沿海开发总体规划，大力发展海洋油气开采及加工业、海洋化工业、海水利用业、海洋生物制药业、海洋产品加工业、船舶及海洋工程装备修造业和以海洋运输为龙头的现代化物流产业，积极发展海洋渔业和滨海旅游业。加强海洋经济综合管理，保持海洋资源的永续利用，努力把唐山建设成为海洋经济强市。

四、积极培育和壮大战略支撑产业

推进经济结构战略性调整，加快产业优化升级，提高自主创新能力，是实现更快更好发展的根本途径。坚持把优化产业结构作为经济工作的首要任务，继续贯彻“存量调强、增量调优”的方针，不断壮大精品钢材、基础能源、优质建材、装备制造和化学工业等优势支柱产业，积极培育高新技术产业，全面振兴服务业，做大做强县域特色产业，努力构筑支撑更快更好可持续发展的产业格局。

优化提升支柱产业。“十一五”期间，全市工业增加值年均增长15%。坚持走新型工业化道路，广泛采用高新技术和先进适用技术改造提升传统产业，引导生产要素向优势产业集中。加快钢铁工业整合重组，推进产品结构向高端、精品、专业化、深加工升级，加快首钢京唐钢铁公司建设，打造国内一流钢铁产业基地。强化能源产业基础性地位，坚持开源、提效并重，加快曹妃甸电厂、冀东油田勘探开发等大项目建设。巩固和提升水泥、建筑卫生陶瓷等名优建材产业优势，加快发展新型优质建材。全面提升装备制造业整体素质，推进水泥和矿山等重型机械、高速电动车组、数控机床等机电一体化开发力度，壮大汽车零部件、自动焊接设备、专用汽车制造、环保机械等新兴产业。化学工业以提高加工深度、延伸产业链为重点，积极打造盐化、煤化和石油化工基地。振兴陶瓷、纺织、食品等劳动密集型传统产业，使之逐步成为优势产业。

培育发展高新技术产业。着力在电子信息、智能机械、新材料、新能源、生物与医药、现代农业等领域，培育形成一批具有较强市场竞争力的优势产业群体。加快重点行业和骨干企业的信息化改造步伐，提升国民经济和社会信息化水平。

加快发展服务业。加速服务业体制、机制、政策和技术创新，创造有利于服务业加快发展的良好环境。坚持市场化、社会化、产业化发展方向，把金融保险、现代物流、信息中介、咨询服务等现代服务业放在优先发展的地位;广泛采用现代经营方式和信息技术，改造提升交通运输、批发零售、餐饮娱乐等传统服务业;鼓励和支持社区服务、商务服务、文体服务等新兴业态加快发展。加强房地产市场调控，引导房地产业健康有序发展。加快旅游资源开发整合，打造旅游品牌，提高旅游业综合竞争力。积极引导和扩大消费需求，增强消费对经济增长的拉动力。

做大做强县域特色产业。依托资源、区位、特色产品优势，培育龙头企业，壮大产业规模，形成各具特色的县域主导产业。积极扶持一批发展潜力较大的特色产业，整合提高，做大做强。充分发挥各类产业园区作用，推动特色产业集约化、集群化发展。打破行政区域界限，优化资源配置，促进互利共赢，共同发展。鼓励支持有条件的县(市、区)跨入全国综合经济实力100强。

加大重点项目建设力度。全社会固定资产投资累计达到4900亿元，其中重点项目投资2100亿元，分别比“十五”增长1.6倍和3.3倍。坚持把项目建设作为优化产业结构、加快经济发展的重要载体，在抓好在建项目的基础上，积极谋划建设一批先进制造业、高新技术产业、现代服务业、社会事业、基础产业和基础设施等符合国家产业政策、产业关联度高、带动能力强的重大项目。重视引进与大项目相配套、延伸产业链条、有利于社会就业的中小项目。深化投融资体制改革，健全多元化投入机制。加强项目管理和引导，实现项目建设与产业布局相协调，与资源条件相适应，与环境容量相匹配，与产业整合相结合，增强可持续发展能力。

五、加快经济增长方式转变

在新的发展阶段，资源环境问题已经成为制约经济社会发展的重要因素。必须切实转变经济增长方式，坚持节约发展、清洁发展、安全发展，使经济增长建立在依靠科技进步、高效利用资源、保护生态环境、提高质量效益的基础上，建设资源节约型、环境友好型社会。

大力发展循环经济。坚持开发节约并重、节约优先，按照“减量化、再利用、资源化”的原则，加快发展节水型农业、节能型工业和低耗型服务业。积极构建农业循环产业链，推进农业生态(园)村建设。在钢铁、建材、能源、化工等重点行业全面推行清洁生产，加快企业节能降耗技术改造，对消耗高、污染重、技术落后的工艺和产品实行强制淘汰。到2010年，工业用水重复利用率提高到85%，固体废物综合利用率提高到70%。在全社会倡导健康文明的生产生活和消费方式，积极创建节约型社会。

加强自然资源和环境保护。坚持保护优先、开发有序，加强水源、土地、森林、海洋、矿产等自然资源的有效管理和保护利用。强化从源头防治污染和保护生态，严格执行项目环境审批，严格实施排放许可和环境影响评价制度，严格实行重点污染源的全面达标排放。加强采煤塌陷区、资源开发密集区生态重建，狠抓滦河、陡河、还乡河等重点流域水污染治理。深化城市环境综合整治，积极防治农村面源污染。搞好环保监管体系、科学支撑体系、资金投入体系和公众参与体系建设，深入开展各种形式的环保创建活动。

加快科技进步。健全完善科技创新体系，打造科技创新基础平台、社会化科技服务平台、科技合作平台、科技人才和科技政策支撑平台，增强自主创新能力。鼓励企业与高校、科研机构建立产学研紧密合作机制，更好发挥企业技术创新和科技投入的主体作用。围绕传统产业改造、科技成果转化和高新技术产业发展，集中力量在重点领域、关键环节进行突破，带动产业结构优化升级。

大力加强人才队伍建设。树立人才资源是第一资源的观念，深入实施“人才强市”战略，逐步使人才队伍整体规模、整体素质和人才结构与转变经济增长方式相适应。多渠道、多形式开展人才智力开发，不断壮大高层次人才队伍，大力培养现代农业和新型工业化亟需的高技能人才和实用人才。进一步完善人才培养、引进、管理、使用配套政策，创新人才评价、激励、分配、保障机制，营造人才辈出、人尽其才的社会氛围和良好环境。

坚持不懈地抓好安全生产。安全生产责任重于泰山，必须坚持安全第一、预防为主、综合治理的方针，切实加大工作力度。严格安全生产责任制，强化企业主体责任。健全安全生产监管体制，综合运用经济、法律和行政手段，狠抓各项措施的落实。建立健全安全生产长效机制，严格企业安全生产准入门槛，进一步加强安全管理制度建设和安全设施建设，搞好职工安全培训。强化执法检查，消除事故隐患，坚决杜绝重特大事故发生，维护人民群众的生命财产安全。

六、建设社会主义新农村

建设社会主义新农村，是我市现代化进程中的重大历史任务。按照生产发展、生活宽裕、乡风文明、村容整洁、管理民主的要求，坚持工业反哺农业、城市支持农村的方针，落实各项惠农政策，加大对农业和农村政策倾斜和资金投入力度，扎实推进新农村建设。

加快建设现代农业。实施科教兴农战略，加强农业基础设施建设，调整产业和产品结构，转变农业增长方式，大力发展高产、优质、高效、生态、安全农业，重视发展外向型农业。实行最严格的耕地保护制度，稳步提高粮食综合生产能力。加快推进乳业、瘦肉型猪、果菜、板栗、花生、水产品六大龙型经济产业升级，积极发展肉牛羊、甘薯、花卉、食用菌和林产品加工等新兴产业。加强特色农产品基地建设，积极培育知名龙头企业和知名品牌，发展各类专业合作组织，提高农业产业化经营水平。2010年，农业产业化经营率达到65%。加快农业标准化建设，提高农业机械化水平，健全农业技术推广、良种繁育和动物防疫、农产品质量安全检测等服务体系。

千方百计增加农民收入。充分挖掘农业内部增收潜力，提高农业综合效益。大力发展农村二、三产业，推动农村劳动力向非农产业转移，拓宽农民增收渠道。积极发展劳务经济，做好农民外出务工就业的组织、指导和服务工作，鼓励和支持农民工劳务输出。加强对农民的劳动就业和引导性职业技能培训，大力培养有文化、懂技术、会经营的新型农民。“十一五”时期，全市转移农村富余劳动力73万人次。

全面深化农村综合改革。稳定和完善以家庭承包经营为基础、统分结合的双层经营体制，建立规范的土地使用权流转机制，发展多种形式的适度规模经营。加快征地制度改革，建立和完善对征地农民的合理补偿机制和失地农民基本生活保障制度。全面取消农业税，搞好乡镇机构、县乡财政管理体制改革，进一步减轻农民负担。推进农村流通体制和金融体制改革。

大力发展农村公共事业。以加强农村基础设施建设为重点，以改善农村人居环境为突破口，按照“经济发展、民主健全、精神充实、环境良好”的要求，继续扎实开展文明生态村创建活动。到2010年，全市70%以上的行政村建成文明生态村。加快发展农村教育事业，增加农村教育投入，重点巩固和提高农村九年义务教育，到2007年对农村义务教育阶段学生全部免收学杂费。积极发展农村文化事业，加强农村公益性文体设施建设。发展完善农村公共卫生和基本医疗体系，抓好县级医院建设和乡镇卫生院、村级卫生室规范化、标准化建设。全面实行新型农村合作医疗制度。完善农村低保制度，逐步提高低保标准。加快区域性敬老院的改造建设步伐。完善和落实“五保户”、困难户等特困群体救助政策。

七、深化体制改革和完善开放型经济格局

坚定不移地推进体制改革，全方位扩大开放，形成更具活力更加开放的体制机制，为实现更快更好发展提供体制保障和强大动力。

在体制改革上，一是深化行政管理体制改革和事业单位改革。加快政府职能转变，提高经济调节和市场监管水平，加强社会管理和公共服务。继续推进政企分开、政资分开、政事分开、政府与市场中介组织分开，进一步精简和规范行政审批。深化财税体制改革，健全公共财政体制，完善预决算和绩效评价机制。分类稳步推进事业单位改革。加快燃气、热力、自来水、污水处理、公交、园林绿化、环卫等市政公用事业单位改制步伐。二是推进国有经济布局调整。大力发展混合所有制经济，推动国有资本向关系国计民生的重要行业和关键领域集中，增强国有经济控制力。深化国有企业改革，健全和完善现代企业制度。积极推进企业联合、整合、并购、重组，培育和发展大型企业集团，支持具备条件的企业上市发展。建立国有资本经营预算制度，健全完善国有资产监管体制，防止国有资产流失。三是大力发展民营经济。全面落实以放宽市场准入、公平待遇、平等竞争为重点的各项政策，支持民营经济进入公用事业、基础设施等法律法规未禁入的行业和领域，鼓励参与国有企业改制改组改造。加强和改进对民营企业的服务和监管，积极推进企业制度创新，鼓励和引导民营企业诚信守法、关爱员工、奉献社会。完善中小企业发展专项资金、投融资和信用担保体系，促进各类所有制中小企业加快发展。四是推进现代市场体系建设。深化流通企业改革，整合流通资源，加快发展连锁经营、物流配送、电子商务等现代流通方式。规范发展资本、土地、技术、产权、劳动力、人才等要素市场。加快社会信用体系建设，规范和维护市场经济秩序。

在开放上，一是积极合理有效地利用外资。大力拓展利用外资领域，改进招商方式，优化投资环境，不断扩大利用外资规模，提高利用外资质量。把引进外资与优化产业结构更好地结合起来，推动利用外资由引进资金为主向全方位引进资金、技术、人才、管理转变，由注重数量为主向更加注重质量转变。加强与世界500强的合资合作，积极争取设立保税区和出口加工区。继续办好唐山中国陶瓷博览会，进一步扩大品牌影响。二是加快转变对外贸易增长方式。优化出口商品结构，培育优势出口企业，提高对外贸易的规模和水平。积极发展加工贸易和服务贸易。重视发挥进口对经济发展的促进作用，增加重要战略资源、关键技术设备进口和先进管理经验引进。深入实施“走出去”战略，支持有条件的企业到境外开展加工贸易，开发能源、资源、对外承包工程和开展劳务输出。三是促进区域经济合作。坚持以我为主、主动服务、承接辐射、促进发展，充分发挥比较优势，进一步扩大京津唐区域经济合作。广泛开展与长三角、珠三角等沿海发达地区的经济技术合作，积极参与西部大开发和东北老工业基地振兴，开辟新的发展空间。

八、建设现代化沿海大城市

深入实施城市化战略，坚持以科学规划为先导，以沿海经济隆起带为支撑，以提高城市现代化水平为重点，加快构建大中小城市和小城镇协调发展的一体化格局，努力把唐山建设成为交通便捷、设施配套、功能完善、环境优美、经济繁荣、适宜人居和创业的现代化沿海大城市。

提高中心城区现代化水平。秉承历史文化传统，创新城市发展理念，提高城市规划建设品位。优化城区产业布局，积极实施“退二进三”。加大公共设施投入力度，努力提高供水、供电、供热、供气和通信能力。建设高效城市路网，完善综合交通体系，大力发展城市公共交通，提高城市交通承载能力。高标准推进机场新区、大南湖生态区等重点区域开发建设。抓好城市综合整治，建设生态城市。

创新城市管理机制。坚持依法、科学、从严、有序管理城市，发挥人民群众参与城市管理的作用。深化城市管理体制综合改革。按照建设现代化沿海大城市的要求，高起点、新思路、大手笔完善规划体系。积极推进城市资源资本化，鼓励民间资本进入基础设施建设领域。把加强城市安全管理放在更加突出位置，建立统一高效的城市应急管理指挥系统，健全突发公共事件应急机制，增强城市防灾、减灾和救灾能力。

加强现代化基础设施建设。在加快城市中心区基础设施建设的同时，按照“统筹规划、适度超前”的原则和生产力布局调整指向，以南部沿海地区为重点，加强综合交通网、电网和水利建设，促进曹妃甸工业区等重点区域的开发。加快承唐、沿海高速公路建设，做好唐曹高速公路前期工作，推进迁(迁安)曹(曹妃甸)、南(南堡)曹(曹妃甸)和遵(遵化南)小(小寺沟)等地方铁路建设，支持国铁干线提速改造，谋划发展城际铁路，融入京津冀都市圈城际轨道交通网。改善供电网络架构，加快城乡电网升级改造，提高电网装备水平和运行质量。完善水利设施，优先保证城市和重点开发区用水。加强信息、通讯、环保、生态设施建设，构建布局合理、功能完善的基础设施体系。

完善现代化城镇布局。加快城市化步伐，建设以中心城市为主体、1个副中心城市、2个中等城市、6个小城市、35个重点镇为主要格局的冀东城市群。在着力增强中心城区辐射带动能力的同时，把加快发展副中心城市和中等城市作为完善城镇体系的战略切入点，促进曹妃甸工业区发展成为新兴港口城区，逐步形成副中心城市;支持迁安、遵化发展成为中等城市;加快发展县城和重点建制镇，培育特色鲜明、功能齐全的小城市和小城镇，形成体系结构完备、功能分工合理的城市体系。

九、促进社会进步与和谐

促进社会和谐，既是全面建设小康社会的重要内容，也是实现更快更好发展的重要保证。要按照“民主法治、公平正义、诚信友爱、充满活力、安定有序、人与自然和谐相处”的要求，扎扎实实地推进和谐唐山建设。

大力发展各项社会事业。优先发展教育事业。深化教育体制改革，优化教育布局和结构，全面推进以素质教育为核心的教育现代化进程。在巩固提高义务教育的基础上，加快普及高中阶段教育，大力发展职业教育，稳步发展高等教育，发展多形式、多层次的再教育，构建全社会终身教育体系，建设学习型社会。努力增加教育投入，健全完善各种教育救助和资助制度。加快发展文化事业和文化产业。落实《唐山市建设文化大市规划纲要》，大力弘扬优秀的冀东地域文化，积极推进文化大市建设。办好中国评剧艺术节等重点文化活动，丰富人民群众文化生活。抓好广电中心、市博物馆及社区公益性文化设施建设。加强文化遗产、重点文物资源的保护。提高全民健康水平。深化医疗卫生体制改革，合理配置卫生资源，完善公共卫生和医疗服务体系，搞好重大疾病预防控制，提高疾病预防、医疗救治水平。整顿药品流通秩序，规范医疗收费标准，切实解决群众看病难、看病贵的问题。广泛开展全民健身运动，积极发展体育产业，提高体育竞技水平。办好十二届省运会。稳定低生育水平，提高出生人口素质。

扩大就业和增加城乡居民收入。把就业摆在经济社会发展更加突出的位置，坚持实施积极的就业政策，充分发挥市场的引导作用，促进多种形式就业。强化政府促进就业的公共服务职能，健全就业服务体系，完善对困难群众的就业援助制度。健全促进就业的有效机制，把国有企业下岗职工再就业扶持政策再延长三年。完善按劳分配为主体、多种分配方式并存的分配制度。建立职工收入与经济效益协调增长机制，逐步提高企业最低工资标准和离退休人员待遇。着力提高低收入者收入水平，扩大中等收入者比重，有效调节过高收入，规范个人收入分配秩序，缓解社会成员收入分配差距扩大趋势。

加强社会保障体系建设。完善城镇职工基本养老、医疗、失业、工伤和生育保险等制度，增加财政投入，依法强化资金征集，进一步健全与经济发展水平相适应、独立于企事业单位之外、资金来源多元化、保障方式多层次、管理服务社会化的社会保障体系。继续完善城镇最低生活保障制度，逐步提高保障标准。加快发展社会福利、老龄和残疾人事业，加强医疗、教育、住房、法律、计划生育等专项救助工作，完善优抚保障机制和社会救助机制。重视保障妇女儿童权益。办好社会慈善事业，提高社会救助能力。

提高精神文明建设水平。加强理想信念教育，大力弘扬以爱国主义为核心的民族精神和以改革创新为核心的时代精神，在全社会形成共同理想和精神支柱。大力弘扬唐山抗震精神，不断丰富和发展抗震精神内涵，使之成为凝聚全市人民力量、加快全面建设小康社会的强大精神动力。加强全民尤其是未成年人的思想道德建设，深入推进社会公德、职业道德和家庭美德教育。以“创五城”活动为载体，继续广泛开展群众性精神文明创建活动，不断提高全民素质和社会文明程度。深入开展国防教育，进一步加强军政军民团结，搞好民兵预备役建设。

加强民主法制建设和维护社会稳定。各级政府要自觉接受人民代表大会及其常委会的法律监督和工作监督，自觉接受人民政协的政治协商、民主监督、参政议政，认真办理人大代表、政协委员的批评、意见、建议和提案。进一步完善向民主党派、工商联、无党派人士、专家学者通报情况和加强联系制度，不断提高决策科学化、民主化水平。加强基层政权和群众性自治组织建设，完善和落实各项公开、公示制度，保证人民群众依法行使选举权、知情权、参与权和监督权。推进依法治市进程，进一步健全与社会主义市场经济、构建文明和谐唐山和改善城市管理相适应的政策法规。扎实开展“五五”普法活动，提高全民法律意识和法律素质。建立健全法律服务体系，切实保障公民和组织的合法权益。全面贯彻党的民族宗教政策，依法管理宗教事务，促进民族团结。妥善处理好新时期人民内部矛盾，进一步加强和改进信访工作，认真解决关系人民群众生产生活的实际问题，维护好人民群众的切身利益。切实加强公共安全管理，提高安全防范水平。落实社会治安综合治理措施，依法严厉打击各种刑事犯罪和经济犯罪，加强隐蔽战线的斗争，深入进行同“法轮功”等邪教组织的斗争。扎实开展创建“诚信平安唐山”活动，努力创造文明法治、稳定和谐、谅解宽容的社会环境。

十、切实加强政府自身建设

全面实现“十一五”的规划目标，完成全面建设小康社会的宏伟蓝图，必须适应新形势、新任务的要求，切实加强政府自身建设，努力开创各项工作新局面。

转变政府职能，建设服务型政府。以科学发展观为指导，进一步推进政府职能转变。在继续履行好经济调节和市场监管职能的同时，更加重视提高社会公共管理水平，更加注重维护社会公平，更加关注民本民生。适应经济社会发展需要，建立健全各种应急机制，提高政府保障公共安全、应对和处置突发事件的能力。加快政府管理模式和管理手段创新，推进电子政务建设和政务公开。深入实施“提速、提质”工程，降低行政成本，提高行政效能，形成行为规范、运转协调、公正透明、廉洁高效的行政管理体制。

坚持行政为民，建设务实政府。牢固树立正确的政绩观，进一步强化民本意识，切实转变工作作风，真正做到想民、亲民、为民。坚持深入基层和实际，注意倾听基层和群众呼声，体察民情，了解民意，尽心竭力为群众做事，维护好人民群众的根本利益。大兴求真务实之风，坚持做事不做“秀”，脚踏实地，埋头苦干，不图虚名，把主要精力集中到抓重点、抓大事、抓主要矛盾上来，用在抓工作落实上来，把各项工作扎扎实实推向前进。

坚持依法行政，建设法治政府。深入贯彻落实《行政许可法》和《公务员法》，进一步提高政府工作人员的法制观念，增强依法行政能力，增强按市场经济规律办事能力，增强运用法律手段管理经济和社会能力。建立健全社情民意反映制度和重大事项社会公示、听证和质询制度，完善行政监督机制，强化对行政权力的制约和监督，把政府行为置于广大人民群众的监督之下，推进政府工作的透明化、公开化、规范化。

深入开展反腐败斗争，建设廉洁政府。认真落实党风廉政建设责任制，坚持标本兼治、综合治理、惩防并举、注重预防的方针，加强理想信念教育、廉洁从政教育和警示教育，增强各级公务员拒腐防变能力，建立健全教育、制度、监督并重的惩治和预防腐败体系。加大查办违纪违法案件力度，严厉惩治各种腐败行为。继续推进民主评议和“阳光行动”，坚决纠正损害群众利益的不正之风，为经济社会发展和社会稳定创造良好环境。

今年是“十一五”规划的开局之年，也是唐山抗震30周年。做好今年的各项工作，具有特殊重要的意义。我们要以纪念抗震30周年为主线，大力弘扬抗震精神，加压奋进，真抓实干，确保经济实力有一个大的增强、社会事业有一个大的进步、城乡面貌有一个大的改善、人民生活有一个大的提高、城市文明有一个大的提升，实现“十一五”的良好开局和扎实起步。

各位代表，回顾过去我们取得了可喜的成就，展望未来我们满怀信心。让我们紧密团结在以胡锦涛同志为总书记的党中央周围，高举邓小平理论和“三个代表”重要思想伟大旗帜，在市委的统一领导下，同心同德，艰苦奋斗，扎实工作，为顺利实现全市国民经济和社会发展第十一个五年规划而努力奋斗!
